# Parity, breastfeeding, and reproductive timing are associated with tumour-infiltrating lymphocytes in hormone receptor-negative breast cancer: a population-based cohort study

**DOI:** 10.1016/j.esmoop.2026.108604

**Published:** 2026-10-21

**Authors:** E. Gabrielsen, A. Wirsing, T. Wilsgaard, I. Thune, H. Frydenberg, T. Lofterød, E. Mortensen, H. Nalwoga, L.A. Akslen, H.S. Haugnes, E.S. Blix

**Affiliations:** 1 Department of Clinical Medicine, UiT–The Arctic University of Norway, Tromsø, Norway; 2 Department of Oncology, University Hospital of North Norway, Tromsø, Norway; 3 Department of Pathology, University Hospital of North Norway, Tromsø, Norway; 4 Department of Medical Biology, UiT–The Arctic University of Norway, Tromsø, Norway; 5 Department of Community Medicine, UiT–The Arctic University of Norway, Tromsø, Norway; 6 Department of Oncology, Oslo University Hospital, Oslo, Norway; 7 Department of Clinical Medicine, Centre for Cancer Biomarkers CCBIO, University of Bergen, Bergen, Norway; 8 Department of Pathology, Makerere University College of Health Sciences, Kampala, Uganda; 9 Department of Oncology and Pathology, Karolinska Institutet, Stockholm, Sweden

**Keywords:** breast cancer, breastfeeding, clinical outcomes, parity, tissue microarray, tumour-infiltrating lymphocytes

## Abstract

**Background:**

Tumour-infiltrating lymphocytes (TILs) are established biomarkers of breast cancer immunogenicity and prognosis. Reproductive events induce durable immunological remodelling of the breast, but whether TIL levels at diagnosis reflect the immunological imprint of these events remains unclear.

**Materials and methods:**

We included women diagnosed with invasive breast cancer between 1978 and 2012 in the Energy Balance and Breast Cancer Aspects throughout Life study, a substudy of the population-based Tromsø Study (Norway). Prediagnostic host factors were obtained. TILs were scored on an ordinal scale (1-4). Associations between host factors and the TIL score were analysed using ordinal logistic regression with interaction terms for hormone receptor (HR) status. Breast cancer-specific mortality was evaluated using Kaplan–Meier methods and Cox regression.

**Results:**

A total of 425 women were included [median age at diagnosis 56.0 years, 360 (87.6%) parous], of whom 84 (19.8%) had HR-negative tumours. Among HR-negative cases, parity [odds ratio (OR) 10.14, 95% confidence interval (CI) 1.97-52.22], longer breastfeeding duration [OR 1.87 per standard deviation (SD), 95% CI 1.10-3.17], and higher age at first birth (OR 1.79 per SD, 95% CI 1.06-3.01) were associated with higher TIL scores, whereas higher mean arterial pressure (OR 0.59 per SD, 95% CI 0.41-0.86) was inversely associated. Significant interactions with HR status were observed for parity and breastfeeding duration. No associations were observed in HR-positive tumours. High-TIL levels (scores 3-4 versus 1-2) were associated with lower breast cancer-specific mortality in HR-negative tumours (adjusted hazard ratio 0.32, 95% CI 0.14-0.72) but not in HR-positive disease (hazard ratio 0.98, 95% CI 0.58-1.68; *P*_interaction_ = 0.023).

**Conclusions:**

These findings suggest that reproductive history and timing may shape the tumour immune microenvironment over the life course. If confirmed in larger studies, TILs may represent an intermediate phenotype linking upstream host characteristics to downstream clinical outcomes in breast cancer.

## Highlights

• Reproductive factors are associated with subtype-specific breast cancer risk and may reflect immune imprinting.
• We studied a population-based cohort with prediagnostic reproductive data, stromal TIL levels, and long-term follow-up.
• Parity, breastfeeding, and reproductive timing were associated with TIL levels only in HR-negative tumours.
• Higher TIL levels were associated with improved BCSS in HR-negative disease only.
• Reproductive history may influence tumour immune phenotype and prognosis in HR-negative breast cancer.

## Introduction

Parity and breastfeeding are well-established modifiers of breast cancer risk.^1^^,^^2^ Their effects are complex and subtype-specific across the life course. Parity is associated with a transient increase in aggressive, often hormone receptor (HR)-negative tumours shortly after childbirth, followed by long-term protection, particularly against HR-positive disease.^1^^,^^3^^,^^4^ Breastfeeding is consistently associated with a reduced risk of HR-negative disease.^1^, ^2^, ^3^, ^4^, ^5^ The biological mechanisms underlying these associations have long remained incompletely understood. In a landmark study, Virassamy et al.^6^ demonstrated that pregnancy and lactation promote long-lasting accumulation of CD8+ tissue-resident memory-like T cells (TRM) within mammary tissue, resulting in enhanced tumour control in murine models. In the same study, breastfeeding was associated with improved survival among women with HR-negative breast cancer and longer breastfeeding duration was linked to higher levels of tumour-infiltrating lymphocytes (TILs).^6^ Together, these findings suggest that reproductive events may imprint long-lasting immune alterations in the breast with relevance for tumour immunogenicity and clinical outcome.

Stromal TILs are a central component of the breast tumour microenvironment and a quantitative marker of host antitumour immune activity. International consensus guidelines have standardised stromal TIL assessment, enabling reproducible evaluation across studies.^7^ Across >2000 publications, TILs have emerged as robust prognostic and predictive biomarkers in breast cancer.^8^ Their clinical relevance is most evident in immunogenic subtypes such as HR-negative disease, in which higher stromal TIL levels are consistently associated with improved survival and enhanced response to systemic therapy.^9^, ^10^, ^11^, ^12^ Accordingly, tumours with abundant immune infiltration are often characterised as immune hot, whereas poorly infiltrated tumours are considered immune cold.^13^

Nevertheless, despite extensive investigation, stromal TILs are almost exclusively assessed at diagnosis and are commonly conceptualised as tumour-intrinsic features of the immune microenvironment, with limited consideration of upstream host determinants that may shape this landscape long before tumour onset. Consistent with experimental and human evidence that reproductive events induce durable T-cell responses and enhance long-term cancer immunosurveillance,^6^ stromal TIL levels at diagnosis may reflect persistent host-immune imprinting established years before tumour initiation rather than tumour-intrinsic processes alone. While reproductive history represents the most biologically plausible determinant of such immune imprinting, other prediagnostic host characteristics, including metabolic status, chronic inflammation, and lifestyle behaviours, may also contribute.^14^, ^15^, ^16^, ^17^

Despite strong biological plausibility, no population-based studies have examined whether prediagnostic reproductive factors are associated with stromal TIL levels at diagnosis. Using data from the population-based Tromsø Study,^18^ we primarily investigated associations between reproductive history and stromal TIL levels in breast cancer. In secondary exploratory analyses, we examined other prediagnostic host factors. Finally, we assessed whether stromal TIL levels in our cohort reproduced established prognostic patterns, both overall and by HR status, in breast cancer. The hypothesised relationships, linking prediagnostic host factors to stromal TIL levels at diagnosis and subsequent breast cancer outcomes, are summarised in Figure 1.

**Figure 1:**
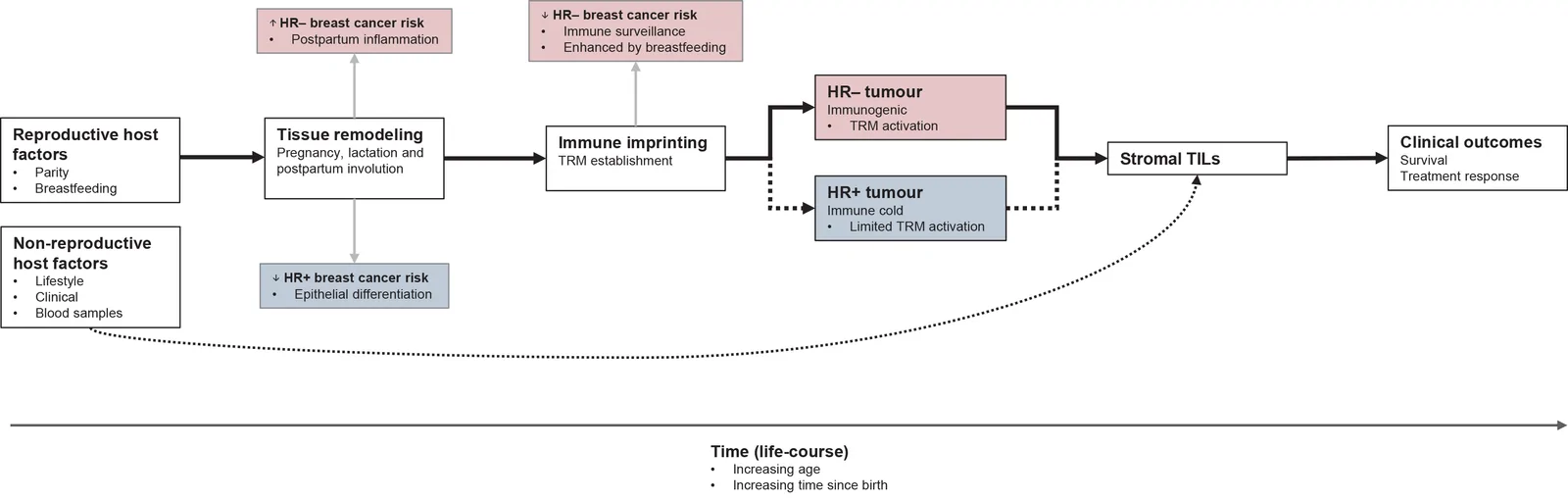
**Conceptual framework linking host factors to the tumour immune microenvironment and clinical outcomes.** The figure illustrates a model in which reproductive host factors induce breast tissue remodelling during pregnancy, lactation, and involution, leading to long-term immune imprinting of the mammary gland via TRMs. These processes may shape the tumour immune phenotype at diagnosis, reflected by stromal TILs. Associations are hypothesized to differ by breast cancer subtype, reflecting differences in tumour immunogenicity. Stromal TILs have established prognostic and predictive clinical relevance in HR-negative disease. Nonreproductive host factors may influence TIL levels through alternative systemic pathways and are depicted separately. The horizontal axis represents the life course, indicating increasing age and time since reproductive events. The influence of immune imprinting is hypothesized to attenuate over time. Solid arrows indicate pathways supported by evidence and dashed arrows indicate weaker or exploratory associations. HR, hormone receptor; TIL, tumour-infiltrating lymphocytes; TRM, tissue-resident memory T cells.

## Materials and methods

### Study design and population

This cohort study was conducted within the Energy Balance and Breast Cancer Aspects throughout Life (EBBA-Life) study, a substudy of the population-based Tromsø Study.^19^, ^20^, ^21^ The Tromsø Study comprises repeated population-based health surveys (Tromsø2-7) conducted among residents of Tromsø municipality,^18^ Norway.

### Prediagnostic host factors

Data on prediagnostic host factors were primarily derived from the Tromsø Study.^18^ Reproductive (parity, age at first and last birth, number of births, and breastfeeding), sociodemographic (married/partner and years of education), and lifestyle (smoking, alcohol consumption, and physical activity) factors were collected through self-administered questionnaires completed by participants before the clinical examination. Clinical measurements (blood pressure, weight, and height) and blood samples [glucose, cholesterol, high-density lipoprotein, triglycerides, and white blood cells (WBCs)] were obtained at the study examination by trained personnel using standardised protocols.^18^ Reproductive host factors were derived from data across all available Tromsø Study survey cycles (Tromsø2-7), whereas sociodemographic, lifestyle, clinical measurements, and blood samples were obtained from the most recent Tromsø Study survey cycle attended before breast cancer diagnosis. Parity was primarily ascertained from questionnaire data, supplemented by medical records when available, yielding information for 411 participants (370 from questionnaires). All other host factors were derived solely from Tromsø Study data. Detailed definitions of host factors are provided in the Supplementary Material (available at https://doi.org/10.1016/j.esmoop.2026.108604).

### Registry linkage

Individual-level record linkage across data sources was carried out using the unique Norwegian personal identification number. Breast cancer cases were identified through the Cancer Registry of Norway.^22^ Information on vital status and cause of death was obtained through linkage with the Norwegian Cause of Death Registry and emigration data were retrieved from the Norwegian Population Registry.^18^^,^^22^

### Histopathology and stromal TIL assessment

Formalin-fixed paraffin-embedded tumour tissue was retrieved from the University Hospital of Northern Norway pathology archives. Representative tumour areas with evaluable tumour-associated stroma were selected on hematoxylin and eosin (H&E)-stained sections by experienced pathologists (E.M., H.N., and L.A.A.), and tissue microarrays (TMAs) were constructed (H.N. and L.A.A.) at the Centre for Cancer Biomarkers CCBIO, University of Bergen, as previously described.^19^^,^^20^

Histopathological and biomarker data were obtained from hospital records by trained physicians (E.G., T.L., and H.F.). Estrogen receptor (ER) and progesterone receptor (PR) were determined by immunohistochemistry (IHC) and human epidermal growth factor receptor 2 status by IHC with *in situ* hybridisation for equivocal cases, in accordance with clinical practice at the time of diagnosis. IHC assessment was also carried out on the TMA material using established protocols.^19^^,^^20^ Tumours were classified as HR-positive if either ER or PR was positive and as HR-negative only if both receptors were negative. In cases of discrepant biomarker information (<10%), the original diagnostic histopathology reports were prioritised.

Stromal TILs were visually assessed on digitised H&E TMA images using a semiquantitative ordinal scoring system, guided by the principles outlined by the International TILs Working Group.^7^ Scoring was carried out independently by two blinded observers (E.G. and A.W.), with discrepancies resolved through consensus or adjudication. Interobserver agreement was assessed using quadratic-weighted Cohen’s κ. Details on the TILs assessment methodology are provided in Supplementary Figure S1 (available at https://doi.org/10.1016/j.esmoop.2026.108604).

### Outcomes

The primary outcome was stromal TIL score, analysed as an ordinal variable (scores 1-4) on H&E-stained tumour tissue. The secondary outcome was breast cancer-specific survival (BCSS), defined as time from breast cancer diagnosis to death from breast cancer.

### Statistical analysis

Descriptive characteristics were summarised as medians with interquartile ranges (IQRs) for continuous variables and counts with percentages for categorical variables.

Associations between host factors and stromal TIL score were assessed using ordinal logistic regression. The proportional odds assumption underlying ordinal logistic regression was evaluated using generalised ordered logistic regression models allowing partial proportional odds; no meaningful violations were observed. Analyses of reproductive host factors were prespecified as primary, whereas analyses of other host factors were considered secondary and exploratory. Adjusted odds ratios (ORs) and 95% confidence intervals (CIs) were reported. All models included HR status and two-way interaction term(s) between HR status and the host factor to evaluate effect modification; HR-specific ORs and 95% CIs were derived from the same model. Reproductive host factors were adjusted for age at diagnosis, whereas prediagnostic host factors were adjusted for age at the study survey cycle and the time interval between the study survey cycle and diagnosis. ORs for continuous variables were presented per standard deviation (SD). Skewed biomarkers (triglycerides, C-reactive protein, and WBC count) were log-transformed before standardisation. Distributions of stromal TIL scores were visualised using stacked bar charts stratified by HR status and across parity and tertiles of breastfeeding duration, age at first birth, and years since first birth.

For BCSS analyses, the TIL score was dichotomised into TIL low (scores 1-2) and TIL high (scores 3-4). Survival curves were estimated using the Kaplan–Meier method and group differences were assessed using the log-rank test. Median follow-up time was calculated using the reverse Kaplan–Meier method. Hazard ratios (HRs) with 95% CIs were estimated using Cox proportional hazards regression, adjusted for age at diagnosis. Effect modification by HR status was evaluated by including a two-way interaction term between TIL category and HR status in the Cox models. Time to event was calculated from the date of breast cancer diagnosis to the date of breast cancer death, defined as breast cancer recorded as the underlying cause of death [International Classification of Disease (ICD)-10 C50; ICD-9 174]. Deaths from other causes, emigration, or the end of follow-up were treated as censoring events. Follow-up was administratively censored on 31 December 2024.

Sensitivity analyses were conducted to assess robustness. To assess potential misclassification of parity, analyses were repeated using questionnaire-derived parity only. As a sensitivity analysis to assess the potential impact of incomplete ascertainment of lifetime breastfeeding duration, analyses were restricted to women whose breastfeeding history was recorded at age ≥40 years, thereby reducing the likelihood of subsequent pregnancies and breastfeeding periods occurring after exposure assessment.

Overall survival (OS) was analysed using the same Kaplan–Meier and Cox modelling strategy as for BCSS. In supplementary exploratory analyses, chronological age at breast cancer diagnosis was modelled separately and in interaction with HR status to characterise age-related differences in stromal TIL score and to inform age adjustment in subsequent analyses. Years since first birth and years since last birth were examined in separate exploratory models without additional age adjustment. Associations between stromal TIL score and body mass index (BMI) at diagnosis, as well as BMI change over time, were also analysed using the same ordinal logistic regression framework.

Additional exploratory analyses restricted to HR-positive tumours examined whether tumour grade (grade 1-2 versus 3) or Ki67 status [low (<20%) versus high (≥20%)] modified the association between breastfeeding duration and stromal TIL score by including interaction terms in ordinal logistic regression models. Exploratory Cox regression analyses in the HR-positive subgroup evaluated whether the association between high and low stromal TIL score and BCSS differed according to tumour grade or Ki67 status and an additional Cox regression model examined BCSS according to histological subtype (invasive lobular versus nonlobular carcinoma).

All analyses were conducted as complete-case analyses. Sample sizes varied across models because of missing prediagnostic host factor data. Statistical tests were two-sided with α = 0.05 and carried out using Stata (version 18; StataCorp LLC, College Station, TX).

## Results

### Study population

In the EBBA-Life cohort, 728 women were diagnosed with invasive breast cancer between 1978 and 2012. Of these, 456 had representative tumour tissue available and were eligible for stromal TIL assessment. After excluding women with stage IV disease, receipt of neoadjuvant systemic therapy, or missing HR status data, 425 women were included in the final cohort (Supplementary Figure S2, available at https://doi.org/10.1016/j.esmoop.2026.108604).

### Patient and tumour characteristics

The study included 425 women with invasive breast cancer, of whom 341 (80.2%) were diagnosed with HR-positive tumours and 84 (19.8%) with HR-negative tumours (Table 1). The median age at diagnosis was 56.0 years (IQR, 48.5-65.6 years) and most women (87.6%) were parous at diagnosis. The median breastfeeding duration was 14 months (IQR, 7-22 months). The distribution of reproductive and prediagnostic host factors was similar across HR subgroups. The median time interval between the last Tromsø Study survey cycle before diagnosis and breast cancer diagnosis was 5.3 years (IQR, 2.6-10.2 years).

**Table 1:** Characteristics of the study population overall and by HR status

Characteristic	*n*	Overall (*n* = 425)	HR-positive (*n* = 341)	HR-negative(*n* = 84)
Clinical characteristics at breast cancer diagnosis	425			
Age at diagnosis (years)		56.0 (48.5-65.6)	57.5 (48.8-66.3)	54.2 (46.9-62.0)
Year of diagnosis		2001 (1994-2007)	2001 (1995-2007)	2000 (1992-2005)
Reproductive host factors^a^				
Parous at diagnosis	411	360 (87.6)	292 (86.9)	68 (90.7)
No. of births^b^	332	2 (2-3)	2 (2-3)	2 (2-3)
Breastfeeding duration (months)^b^	293	14 (7-22)	13 (7-22)	14 (6-24)
Age at first birth (years)^b^	297	23 (21-27)	24 (21-27)	23 (21-26)
Age at last birth (years)^b^	297	30 (27-35)	30 (27-35)	30 (25-36)
Years since first birth at diagnosis^b^	297	34 (25-43)	34 (25-44)	33 (21-43)
Years since last birth at diagnosis^b^	297	28 (19-37)	27 (19-37)	28 (14-37)
Prediagnostic host factors^c^				
Sociodemographic				
Married/partner	337	188 (55.8)	151 (55.3)	37 (57.8)
Years of education	361	11 (9-15)	11 (9-15)	11 (9-15)
Lifestyle				
Current smoking	379	145 (38.3)	117 (39.1)	28 (35.0)
No current alcohol consumption	319	43 (13.5)	30 (11.7)	13 (21.0)
Physical activity				
Low	359	84 (23.4)	68 (23.8)	16 (21.9)
Medium		210 (58.5)	164 (57.3)	46 (63.0)
High		65 (18.1)	54 (18.9)	11 (15.1)
Clinical				
MAP (mmHg)	379	92.3 (84.7-103.0)	92.3 (84.7-102.0)	92.5 (85.3-106.8)
BMI (kg/m^2^)	377	23.9 (21.8-27.0)	23.9 (21.5-26.9)	24.1 (22.0-27.3)
Blood samples				
Glucose (mmol/l)	188	4.9 (4.6-5.5)	4.9 (4.6-5.5)	5.0 (4.6-5.5)
Cholesterol (mmol/l)	377	5.8 (5.1-6.8)	5.8 (5.1-6.8)	5.8 (5.0-6.8)
HDL (mmol/l)	377	1.6 (1.4-1.9)	1.6 (1.4-1.9)	1.6 (1.4-2.0)
Triglycerides (mmol/l)	377	1.1 (0.78-1.6)	1.1 (0.78-1.6)	1.1 (0.80-1.5)
WBC count (×10^9^/l)	243	6.5 (5.4-7.8)	6.5 (5.4-7.9)	6.5 (4.9-7.5)
Timing of exposure assessment				
Years between the last study survey cycle and diagnosis	379	5.3 (2.6-10.2)	5.7 (2.6-10.8)	4.8 (2.6-6.5)

Continuous variables are presented as median (IQR) and categorical variables as *n* (%). *n* may vary across characteristics due to missing data.

BMI, body mass index; HDL, high-density lipoprotein; HR, hormone receptor; IQR, interquartile range; MAP, mean arterial pressure; WBC, white blood cell.

a Reproductive variables were derived using all available information across Tromsø Study surveys (Tromsø2-7): information on age at births and breastfeeding was available from Tromsø4 onwards. Parity was supplemented with medical records, increasing completeness from 370 to 411 women.

b Parous women only.

c Sociodemographic, lifestyle, clinical, and blood-based variables were obtained from the most recent Tromsø Study survey cycle (Tromsø2-6) before breast cancer diagnosis.

Interobserver agreement for stromal TIL scoring was κ = 0.70 (quadratic-weighted, *P* < 0.001). Compared with HR-positive tumours, HR-negative tumours were more frequently high grade, had higher proliferative activity (Ki-67 ≥ 20%), and exhibited higher stromal TIL scores (Supplementary Table S1 available at https://doi.org/10.1016/j.esmoop.2026.108604). Most tumours had intermediate stromal TIL scores (scores 2-3: 81.4%), whereas low (score 1) and high (score 4) infiltration were less common (9.9% and 8.7%, respectively).

### Host factors and TIL levels

In the prespecified primary analyses, several reproductive host factors were associated with stromal TIL score, with interactions by HR status (Table 2). Parity (HR-positive: OR 1.27, 95% CI 0.69-2.34, versus HR-negative: OR 10.14, 95% CI 1.97-52.22, *P*_interaction_ = 0.020), longer breastfeeding duration (HR-positive: OR 0.88 per SD increase, 95% CI 0.68-1.13, versus HR-negative: OR 1.87, 95% CI 1.10-3.17, *P*_interaction_ = 0.011), and higher age at first birth (HR-positive: OR 1.04, 95% CI 0.81-1.33, versus HR-negative: OR 1.79, 95% CI 1.06-3.01, *P*_interaction_ = 0.066) showed associations confined to HR-negative tumours. Estimates for parity in HR-negative tumours were based on a limited number of nulliparous women (*n* = 7). Distributions of TIL scores by selected reproductive host factors and HR status are shown in Figure 2.

**Table 2:** Associations between host factors and stromal TIL score by HR status^a^

Host factors	*n*	HR-positive, OR (95% CI)	HR-negative, OR (95% CI)	*P*_interaction_
Reproductive^b^				
Parous at diagnosis, parous versus nulliparous (reference)^c^	411	1.27 (0.69-2.34)	**10.14 (1.97-52.22)**	**0.020**
No. of births, per SD increase^d^	332	1.00 (0.78-1.29)	1.05 (0.67-1.64)	0.858
Breastfeeding duration (months),^e^ per SD increase^d^	293	0.88 (0.68-1.13)	**1.87 (1.10**-**3.17)**	**0.011**
Age at first birth (years), per SD increase^d^	297	1.04 (0.81-1.33)	**1.79 (1.06-3.01)**	0.066
Age at last birth (years), per SD increase^d^	297	1.05 (0.81-1.37)	1.49 (0.96-2.31)	0.182
Years since first birth at diagnosis, per SD increase^d^	297	0.75 (0.51-1.37)	0.50 (0.24-1.05)	0.165
Years since last birth at diagnosis, per SD increase^d^	297	0.81 (0.48-1.38)	0.55 (0.29-1.06)	0.178
Prediagnostic^f^				
Lifestyle				
Smoking, current versus never (reference)	379	0.81 (0.52-1.27)	0.54 (0.22-1.32)	0.429
Current alcohol consumption, no versus yes (reference)	319	1.37 (0.65-2.87)	1.56 (0.45-5.42)	0.857
Physical activity	359			0.472
Low (reference)		Reference	Reference	
Medium		0.79 (0.46-1.37)	1.51 (0.52-4.32)	
High		0.74 (0.37-1.48)	1.75 (0.42-7.21)	
Clinical, per SD increase				
MAP (mmHg)	379	0.88 (0.68-1.14)	**0.59 (0.41-0.86)**	0.071
BMI (kg/m^2^)	377	0.88 (0.69-1.12)	0.90 (0.59-1.38)	0.928
Blood samples, per SD increase				
Glucose (mmol/l)	188	0.75 (0.55-1.01)	0.81 (0.37-1.77)	0.838
Cholesterol (mmol/l)	377	0.98 (0.77-1.26)	0.86 (0.56-1.31)	0.592
HDL (mmol/l)	377	0.95 (0.76-1.18)	1.04 (0.66-1.65)	0.707
Triglycerides (mmol/l)^g^	377	0.94 (0.76-1.18)	1.10 (0.67-1.81)	0.572
WBC count (×10^9^/l)^g^	243	0.84 (0.64-1.11)	1.39 (0.76-2.54)	0.139

*n* may vary across host factors due to missing data.

Bold values indicate statistically significant associations (*P* < 0.05).

BMI, body mass index; CI, confidence interval; HDL, high-density lipoprotein; HR, hormone receptor; MAP, mean arterial pressure; OR, odds ratio; SD, standard deviation; TIL, tumour-infiltrating lymphocyte; WBC, white blood cell.

a ORs (95% CIs) were estimated using ordinal logistic regression of stromal TIL score (1-4). All models included HR status and two-way interaction terms with host factors; ORs are presented by HR status. For categorical variables with three levels, a joint Wald test of the interaction terms was used to derive the *P* value for interaction.

b Adjusted for age at diagnosis.

c Estimates for HR-negative tumours are based on a limited number of nulliparous women (*n* = 7) and should be interpreted with caution.

d Parous women only.

e Total breastfeeding duration across all children.

f Adjusted for age at study survey cycle and the time between study survey cycle and diagnosis.

g Log-transformed before standardisation.

**Figure 2:**
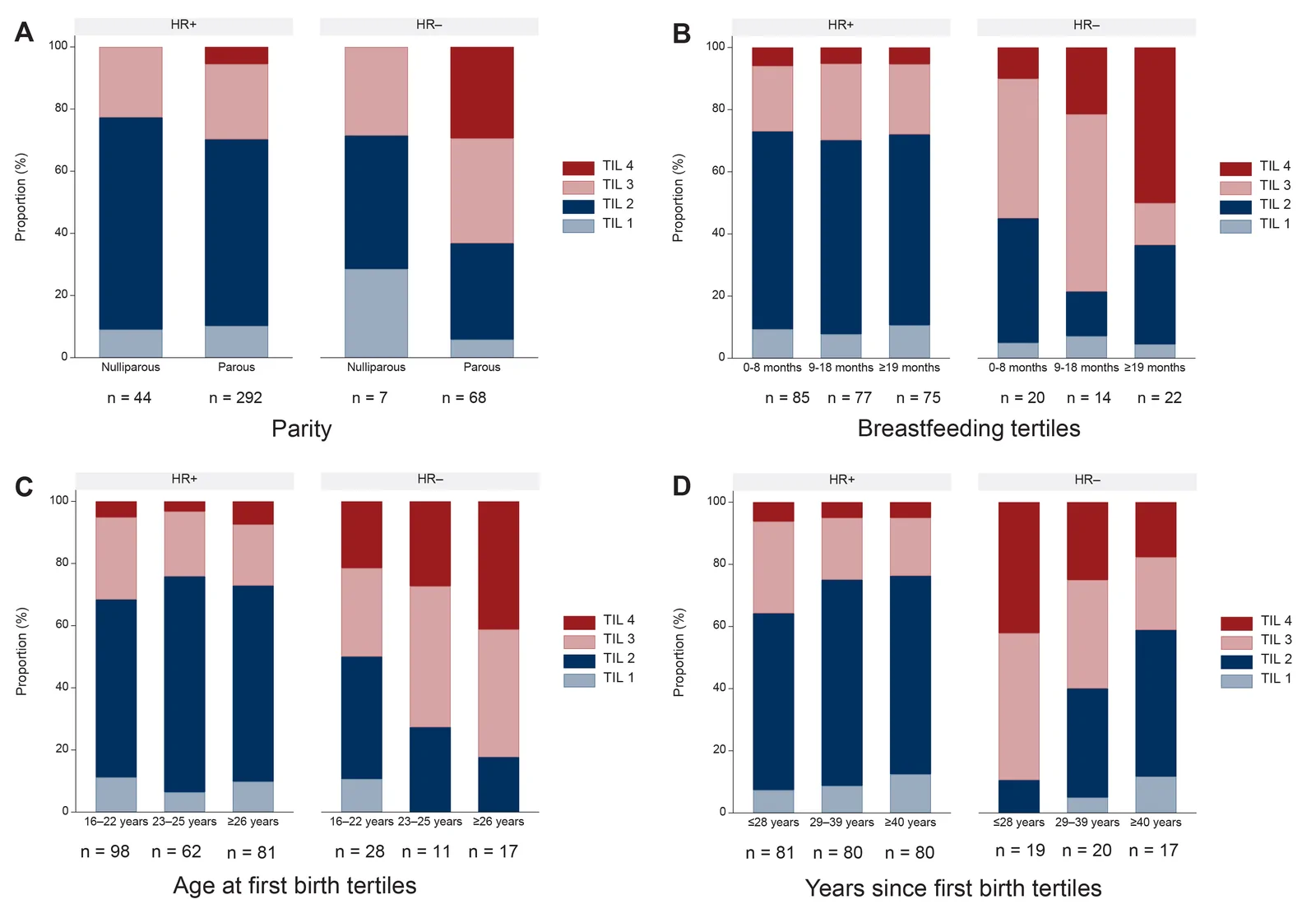
**Stromal TIL score by reproductive host factors and HR status.** Stacked bar charts show the distribution of stromal TIL scores (1-4) within HR-positive and HR-negative tumours according to parity status at diagnosis (A), tertiles of total lifetime breastfeeding duration (B), tertiles of age at first birth (C), and tertiles of years since first birth (D). HR, hormone receptor; TIL, tumour-infiltrating lymphocytes.

In secondary analyses of other prediagnostic host factors, mean arterial pressure (MAP) was inversely associated with stromal TIL score (HR-positive: OR 0.88, 95% CI 0.68-1.14, versus HR-negative: OR 0.59, 95% CI 0.41-0.86, *P*_interaction_ = 0.071) only in HR-negative tumours. No associations were observed for BMI, lifestyle factors, or circulating metabolic and inflammatory biomarkers.

### TIL levels and survival

We next assessed whether stromal TIL levels were associated with BCSS. Baseline characteristics according to TIL low (score 1-2) and high (score 3-4) are presented in Supplementary Table S2 (available at https://doi.org/10.1016/j.esmoop.2026.108604). During a median follow-up of 18.9 years (95% CI 17.4-19.7 years), 87 breast cancer-specific deaths occurred. In the overall cohort, BCSS did not differ between patients in the low- and high-TIL group (log-rank *P* = 0.472) (Figure 3A). The findings were consistent in Cox regression analyses (breast cancer-specific death: adjusted HR 0.81, 95% CI 0.52-1.28). Among HR-positive tumours, BCSS was similar between TIL groups (log-rank *P* = 0.961) (Figure 3B), with an adjusted HR of 0.98 (95% CI 0.58-1.68). Among HR-negative tumours, BCSS was higher in the high-TIL group than in the low-TIL group (log-rank *P* = 0.016) (Figure 3C). The 10-year BCSS was 82.9% (95% CI 68.7% to 91.1%) in the high-TIL compared with 59.5% (95% CI 40.5% to 74.2%) in the low-TIL group and the 20-year BCSS was 80.7% (95% CI 66.1% to 89.4%) versus 55.6% (95% CI 36.5% to 71%), respectively. Among HR-negative tumours, the adjusted HR for breast cancer death was 0.32 (95% CI 0.14-0.72) for high- versus low-TIL levels, with a significant interaction by HR status (*P*_interaction_ = 0.023). Notably, among HR-negative tumours with the highest TIL score (TIL score = 4, *n* = 21), we observed only one event for breast cancer death.

**Figure 3:**
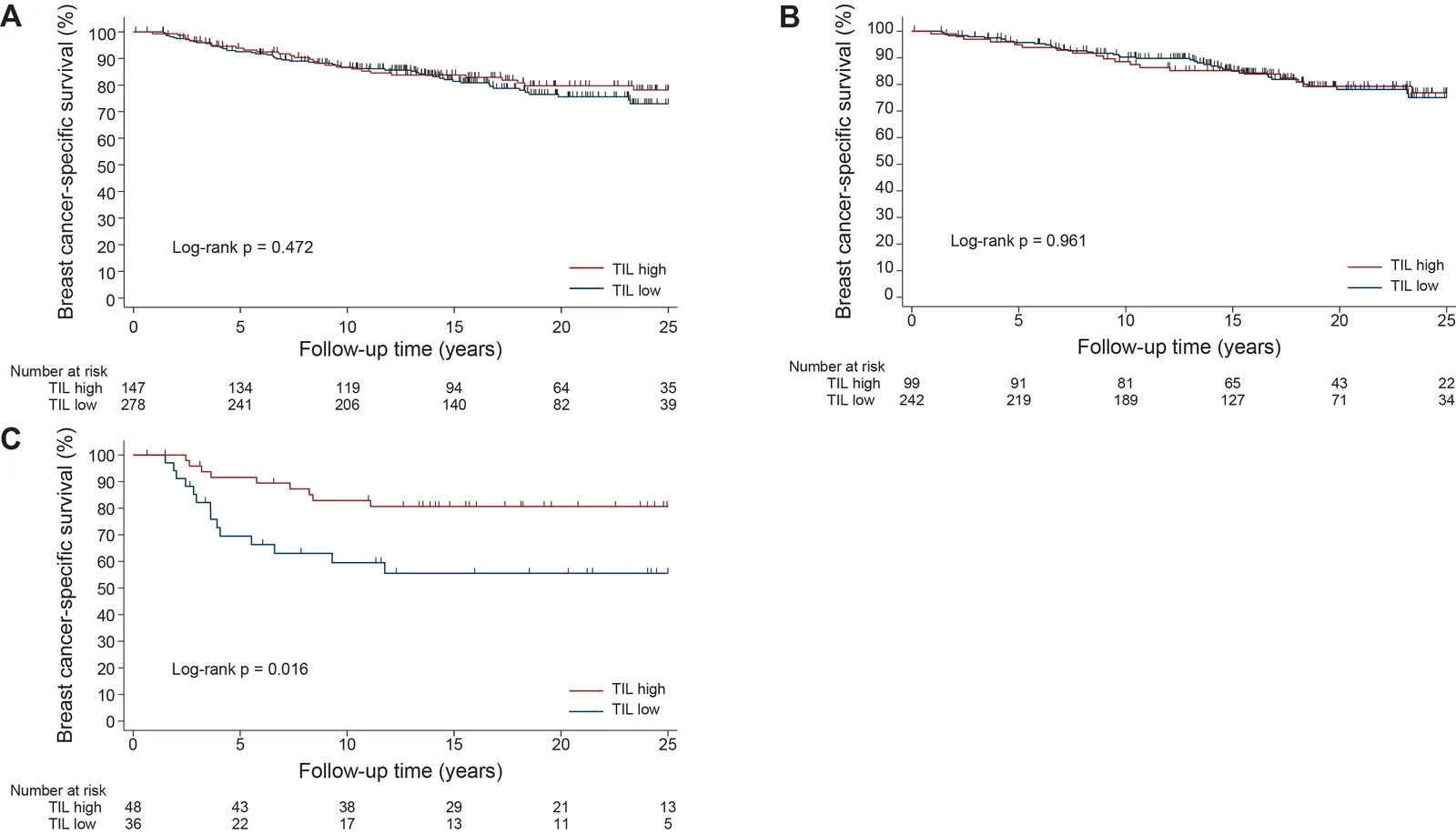
**Kaplan–Meier estimates of breast cancer-specific survival by stromal TIL group.** Survival curves are shown for the overall cohort (A), HR-positive tumours (B), and HR-negative tumours (C). Stromal TIL score was dichotomized as low (scores 1-2) or high (scores 3-4). HR, hormone receptor; TIL, tumour-infiltrating lymphocytes.

### Sensitivity, supplementary, and exploratory analyses

Sensitivity analyses produced similar estimates for parity and breastfeeding duration, and results remained consistent when OS was used as the endpoint. In additional unadjusted analyses, longer time since the first and last birth was associated with lower TIL scores across both HR subtypes. Age at diagnosis was associated with lower stromal TIL scores in HR-positive disease, with similar but less precise estimates in HR-negative disease. No associations were observed for BMI at diagnosis or for changes in BMI over time. Detailed results are provided in the Supplementary Material (available at https://doi.org/10.1016/j.esmoop.2026.108604).

Additional exploratory analyses restricted to HR-positive tumours examined whether tumour grade or Ki67 status modified the association between breastfeeding duration and stromal TIL score. There was no statistically significant evidence of interaction. However, the estimated association was stronger in grade 3 than in grade 1-2 tumours (interaction OR 1.79, 95% CI 0.72-4.47) and in Ki67-high than in Ki67-low tumours (interaction OR 1.61, 95% CI 0.63-4.07). ER-low tumours could not be reliably examined because ER expression was reported in broad percentage categories, with only six tumours having a reported ER expression of 10%. Exploratory survival analyses in the HR-positive subgroup showed no evidence that the association between high versus low stromal TIL score and BCSS differed according to tumour grade (interaction HR 1.92, 95% CI 0.45-8.25) or Ki67 status (interaction HR 1.31, 95% CI 0.41-4.24). In an additional exploratory analysis, BCSS did not differ significantly between invasive lobular and nonlobular carcinomas (HR 1.32, 95% CI 0.76-2.31).

## Discussion

In this population-based cohort study, prediagnostic reproductive host factors were associated with stromal TIL levels at diagnosis. Parity, higher age at first birth, and longer breastfeeding duration were each associated with higher TIL levels, with significant interactions by HR status for parity and breastfeeding duration. In turn, higher stromal TIL levels were associated with improved BCSS, with significant interaction by HR status. Effects were restricted to HR-negative tumours.

Apart from MAP, nonreproductive host factors were not significantly associated with stromal TIL levels in secondary analyses. Among HR-negative tumours, effect estimates were imprecise but generally directionally consistent. This pattern may suggest that lifestyle and clinical exposures influence tumour immunity through broader systemic modulation,^23^ rather than breast-specific immune imprinting for reproductive exposures.

When reproductive history was examined in greater detail among HR-negative tumours, parity emerged as the primary reproductive exposure associated with higher stromal TIL levels, suggesting that childbirth may establish a baseline immune imprint within the breast. Among parous women, both cumulative exposure and timing-related factors further modified the degree of infiltration. Longer breastfeeding duration was associated with higher TIL levels, consistent with a dose–response effect, whereas the number of births was not, indicating a threshold rather than a cumulative relationship. Older age at first birth was likewise associated with higher TIL levels. In contrast, longer intervals since the first and last childbirth showed inverse associations, consistent with the gradual attenuation of this imprint over time. Although associations for these interval measures weakened after adjustment for age at diagnosis, likely reflecting collinearity in timing-related measures, the consistent direction of effects supports a time-dependent process. Effect estimates for older age at diagnosis were similar across HR subtypes, which may reflect general immunosenescence, an age-related decline in immune competence.^24^ Together, these findings suggest that stromal immune infiltration reflects both global age-related immune decline and a subtype-specific reproductive immune imprint shaped by reproductive exposures and diminishing with time since childbirth.

These observations can be interpreted in the context of the immunological remodelling that accompanies pregnancy, lactation, and postpartum mammary gland involution.^25^, ^26^, ^27^, ^28^ Immune cells also contribute to normal mammary gland development and epithelial morphogenesis.^29^ Involution has been linked to a transiently increased risk of aggressive breast cancer subtypes and poorer outcomes after childbirth,^1^^,^^3^^,^^26^^,^^27^ whereas breastfeeding attenuates this risk by promoting immune resolution and limiting the protumourigenic inflammatory milieu of the postpartum breast.^1^^,^^2^^,^^5^^,^^28^,^30^^,^^31^ Such processes may leave durable immunological imprints in the breast, whereby parity may establish TRMs, with breastfeeding further expanding this pool and thereby enhancing tumour surveillance. These TRM populations correlate with TIL density and may facilitate recruitment and retention of circulating effector T cells rather than mediating tumour control in isolation.^6^ In this framework, the reproductive exposures associated with higher TIL levels likely reflect this underlying immune imprint as captured by routine histopathology. Extending the findings of Virassamy et al.^6^, our results further suggest that reproductive timing is a critical determinant of this imprinting effect. The marked contrast between HR subtypes indicates that host-derived immune programming becomes clinically apparent primarily in immunogenic disease, where pre-existing tissue-resident immunity can be functionally engaged, manifesting as higher TIL levels and improved survival. Consistent with prior reports, stromal TILs in our cohort were prognostic in HR-negative but not in HR-positive tumours.^9^, ^10^, ^11^, ^12^ Together, these observations support tumour immune contexture as a potential intermediate phenotype linking reproductive history to subtype-specific breast cancer risk and prognosis. Recent research has emphasised the importance of considering HR status, tumour cell proliferation, and the immune microenvironment together when characterising breast cancer subtypes.^32^ Our exploratory analyses in HR-positive tumours found no statistically significant interactions between breastfeeding duration and tumour grade or Ki67 status in relation to TIL levels, possibly due to limited statistical power, and these relationships should be investigated in larger cohorts.

More broadly, our results have implications for how tumour immunity is conceptualised. Immune infiltration is often viewed as a contemporaneous response to tumour-intrinsic biology alone yet our findings indicate that it may also reflect host-immune programming established years before diagnosis, helping to explain heterogeneity in TIL levels and clinical outcomes. Because many tissues undergo repeated cycles of remodelling and inflammation^23^ and immune infiltration is recognised as a favourable prognostic biomarker across multiple solid tumours,^9^^,^^10^ similar host-immune mechanisms may operate beyond the breast and warrant investigation in other cancers. At a population level, these observations further suggest that life-course exposures may shape tumour immunity in modifiable ways, with potential implications for cancer prevention and long-term risk stratification.

Key strengths of this study include the population-based design within the Tromsø Study, repeated prediagnostic assessments that minimise reverse causation, linkage to nationwide registries ensuring virtually complete follow-up,^18^^,^^22^ and standardised stromal TIL assessment,^7^ which enhanced reproducibility and comparability across studies. Previous studies have demonstrated that TMA-based immune biomarker assessment retains prognostic value in large retrospective breast cancer cohorts.^33^ Limitations include the observational design; potential residual confounding; use of an ordinal TIL scale; lack of characterisation of specific immune cell subsets; lack of information on menstrual cycle phase at the time of tumour sampling, which may influence the tumour microenvironment^34^; and limited statistical power for analyses restricted to HR-negative tumours. Confirmation of the HR-negative findings in larger studies is warranted.

### Conclusions

Stromal TIL levels in breast cancer partly reflect long-term host characteristics established years before diagnosis, with effects confined to HR-negative disease. By linking prediagnostic host factors to tumour immune infiltration at diagnosis and subsequent BCSS, this study supports a life-course perspective on tumour immunogenicity and identifies reproductive history and timing as previously underexplored host determinants of the breast tumour immune phenotype. If confirmed in larger studies, these findings may have implications for breast cancer prevention, risk stratification, and the design of future clinical trials.

## Ethical approval

The Energy Balance and Breast Cancer Aspects throughout Life and Tromsø Study protocols, including linkage to national health registries, were approved by the Regional Committee for Medical and Health Research Ethics (REK; 2015/599 and 2014/940). Participation was voluntary. From Tromsø4 onwards, all participants provided written informed consent for use of questionnaire data, biological samples, and linkage to national health registries. Earlier surveys were conducted in accordance with the ethical standards of the time.

## Data availability

Data are not publicly available because of national ethical and privacy regulations. Deidentified data may be made available upon reasonable request through the Tromsø Study data repository, subject to approval by the study’s data access committee and relevant regulatory authorities.

## Funding

The present study was supported by grants from the 10.13039/501100007137Northern Norway Regional Health Authority [grant numbers SFP1273-16 and HNF1529-20], the 10.13039/501100005416Norwegian Research Council [grant number 213997], South-East Norwegian Health Authority [grant number 2811507], The Norwegian Extra Foundation for Health and Rehabilitation [grant number 2013/FOM 5628], and The 10.13039/100008730Norwegian Cancer Society [grant number 2012/2/0054]. The Tromsø Study was supported by the 10.13039/501100005416Research Council of Norway, the Norwegian Council on Cardiovascular Disease, the 10.13039/501100007137Northern Norway Regional Health Authority, the University of Tromsø, the 10.13039/100009471Norwegian Foundation for Health and Rehabilitation, and the Odd Berg Research Foundation.

## Declaration of AI and AI-assisted technologies in the writing process

During the preparation of this work, the authors used ChatUiT in order to assist with language editing and grammar. After using this tool, the authors reviewed and edited the content as needed and take full responsibility for the content of the published article.
