# Supplementary material for: Parity, breastfeeding, and reproductive timing are associated with tumour-infiltrating lymphocytes in hormone receptor-negative breast cancer: a population-based cohort study
Source: ESMO Open. 2026 Oct 21;11(11):108604. doi: 10.1016/j.esmoop.2026.108604 (PMC13717872; doi:10.1016/j.esmoop.2026.108604)
Supplement: Supplementary file 1 [file mmc1.docx]

**Supplementary Material**

**Materials and methods**

*Definition of reproductive host factors*

Parity at breast cancer diagnosis was classified using information on childbirth reported across Tromsø Study survey cycles and the timing of each survey cycle relative to diagnosis. Women were classified as parous if they reported at least one birth in any survey cycle prior to breast cancer diagnosis. In addition, women aged 40 years or older at diagnosis who reported at least one birth in any survey cycle were classified as parous. Nulliparity was conservatively defined among women reporting zero births at age 40 years or older in any survey cycle. In addition, women reporting zero births in survey cycles occurring after diagnosis were also classified as nulliparous. Missing parity data among women aged ≥40 years at diagnosis were supplemented using medical records, increasing completeness from 370 to 411.

Among parous women, age at first and last birth were calculated as the difference between the corresponding birth year and the woman’s year of birth. Years since first and last birth were calculated as age at diagnosis minus age at first and last birth, respectively. The number of births was defined as the maximum number reported across all available Tromsø Study survey cycles. For each child, breastfeeding duration was defined as the highest number of months reported across all available survey cycles, assuming this reflected completed breastfeeding. Total lifetime breastfeeding duration was calculated by summing breastfeeding duration across all children.

*Definition of pre-diagnostic host factors*

Marital status was originally recorded as single, married/registered partnership, widowed, divorced, or separated, and was dichotomized as married or with partner versus all other categories. Leisure-time physical activity and years of education were harmonised across survey cycles using survey cycle-specific questionnaire items; physical activity was categorised as low, moderate, or high, and education was treated as a continuous variable (years). Smoking status was categorised as never, former, or current smoker. Alcohol consumption was dichotomised (yes/no). Mean arterial pressure (MAP) was calculated as (systolic blood pressure + 2 × diastolic blood pressure)/3. BMI was calculated as weight divided by height squared.

All pre-diagnostic host factors were derived from the Tromsø Study survey cycle closest in time before breast cancer diagnosis. All variables were obtained from this same survey cycle to ensure temporal alignment. Participants without any pre-diagnostic survey cycle had missing values for all pre-diagnostic variables. Because not all variables were collected in every survey cycle, additional variable-specific missingness occurred. Age at the selected survey cycle and the time interval (years) between data collection and diagnosis were calculated from the same survey cycle.

*Stromal TIL scoring*

Stromal tumor-infiltrating lymphocytes (TILs) were defined as the proportion of tumour-associated stromal area within the borders of the invasive tumor occupied by mononuclear inflammatory cells, excluding areas of necrosis, in situ components, and extra-tumoural stroma ^1^. Intratumoural TILs were not assessed. Given the limited tissue area inherent to tissue microarray-based assessment, stromal TILs were evaluated using a semi-quantitative ordinal scoring system rather than continuous percentage estimates. Each representative core was assigned an ordinal score ranging from 1 to 4 (1 = none/scant, 2 = low, 3 = moderate, and 4 = high infiltration). A core was considered representative if at least 50% of the tissue area was intact, and invasive tumor cells comprised at least 10% of the total core area. TIL scoring was performed independently by two observers (EG, AW), blinded to host factors, clinical data, and outcomes. Discrepancies were resolved by consensus or, when necessary, adjudicated by a senior pathologist (EM). If adjudication remained inconclusive or fewer than two representative cores were available, evaluation was performed on whole H&E-stained sections.

**Results**

In sensitivity analyses restricted to questionnaire-derived parity data only (n = 370), estimates were similar to those in the main analysis (HR+: OR 1.15, 95% CI 0.59–2.26; HR–: OR 12.95, 95% CI 2.03–82.38; p for interaction 0.016). In analyses restricted to women with breastfeeding information recorded at age ≥40 years (n = 283), effect estimates for breastfeeding duration were likewise similar to those in the main analysis (HR+: OR 0.89 per SD increase, 95% CI 0.68–1.15; HR–: OR 1.98 per SD increase, 95% CI 1.15–3.40; p for interaction 0.009). For the overall survival (OS) analyses, 228 deaths occurred during follow-up (figure S3). Higher TIL levels (score 3-4 vs score 1-2) were associated with improved survival in HR– tumours, but not in HR+ tumours (HR+: HR 0.87, 95% CI 0.62–1.22 vs HR–: HR 0.40, 95% CI 0.23–0.72), with a significant interaction by hormone receptor status (p for interaction 0.026).

In supplementary unadjusted analyses, longer time since first birth was inversely associated with TIL score, most strongly in HR– tumours (HR–: OR 0.48 per SD increase, 95% CI 0.28–0.82), with a weaker association in HR+ disease (HR+: OR 0.73 per SD increase, 95% CI 0.56–0.94; p for interaction 0.17). Similarly, longer time since last birth was inversely associated with TIL score in both HR– and HR+ tumours (HR+: OR 0.73 per SD increase, 95% CI 0.56–0.95; HR–: OR 0.51 per SD increase, 95% CI 0.31–0.83; p for interaction 0.19). Increasing age at diagnosis showed comparable inverse associations across HR subtypes (HR+: OR 0.73 per SD increase, 95% CI 0.59–0.90; HR–: OR 0.71 per SD increase, 95% CI 0.45–1.12; p for interaction 0.91). Neither BMI at diagnosis (n = 362) nor change in BMI over time (n = 335) was associated with stromal TIL score, with similar null estimates across HR subtypes and no evidence of interaction by HR status (all p for interaction >0.60).

**Table S1 Tumour characteristics overall and by hormone receptor status**

| **Characteristic** | | **n** | **Overall**  **(n = 425)** | **HR+**  **(n = 341)** | **HR–**  **(n = 84)** |
| --- | --- | --- | --- | --- | --- |
| **Histologic type** | 421 | |  |  |  |
| Invasive carcinoma NST |  | | 340 (80.8%) | 269 (79.8%) | 71 (84.5%) |
| Invasive lobular carcinoma |  | | 58 (13.8%) | 55 (16.3%) | 3 (3.6%) |
| Other |  | | 23 (5.5%) | 13 (3.9%) | 10 (11.9%) |
| **Histological grade** | 353 | |  |  |  |
| 1 |  | | 114 (32.9%) | 111 (38.7%) | 3 (4.5%) |
| 2 |  | | 173 (49.0%) | 149 (51.9%) | 24 (36.4%) |
| 3 |  | | 66 (18.7%) | 27 (9.4%) | 39 (59.1%) |
| **T-status** | 400 | |  |  |  |
| pT1 |  | | 225 (56.2%) | 195 (57.1%) | 40 (52.6%) |
| pT2 |  | | 145 (36.2%) | 114 (35.2%) | 31 (40.8%) |
| pT3-4 |  | | 30 (7.5%) | 25 (7.7%) | 5 (6.6%) |
| **N-status** | 409 | |  |  |  |
| pN0 |  | | 277 (67.7%) | 229 (69.2%) | 48 (61.5%) |
| pN1 |  | | 101 (24.7%) | 79 (23.9%) | 22 (28.2%) |
| pN2-3 |  | | 31 (7.6%) | 23 (6.9%) | 8 (10.3%) |
| **Stage** | 422 | |  |  |  |
| I |  | | 193 (45.7%) | 161 (47.5%) | 32 (38.6%) |
| II |  | | 187 (44.3%) | 149 (44.0%) | 38 (45.8%) |
| III |  | | 42 (10.0%) | 29 (8.6%) | 13 (15.7%) |
| **Ki-67** | 384 | |  |  |  |
| <20% |  | | 264 (68.8%) | 246 (80.1%) | 18 (23.4%) |
| ≥20% |  | | 120 (31.2%) | 61 (19.9%) | 59 (76.6%) |
| **HER2 status** | 411 | |  |  |  |
| Negative |  | | 343 (83.5%) | 292 (87.5%) | 51 (65.4%) |
| Positive |  | | 68 (16.5%) | 41 (12.3%) | 27 (34.6%) |
| **Stromal TIL score** | 425 | |  |  |  |
| 1 (lowest) |  | | 42 (9.9%) | 35 (10.3%) | 7 (8.3%) |
| 2 |  | | 236 (55.5%) | 207 (60.7%) | 29 (34.5%) |
| 3 |  | | 110 (25.9%) | 83 (24.3%) | 27 (32.1%) |
| 4 (highest) |  | | 37 (8.7%) | 16 (4.7%) | 21 (25.0%) |

All variables are presented as n (%).

n may vary across characteristics due to missing data

Abbreviations: HR+, hormone receptor-positive; HR–, hormone receptor-negative; n, number of non-missing; NST, no special type; TIL, tumour-infiltrating lymphocytes.

**Table S2 Selected reproductive, pre-diagnostic host, and tumour characteristics by stromal TIL category (low vs high)**

| **Characteristic** | **n** | **TIL low**  **(n = 278)** | **TIL high**  **(n = 147)** |
| --- | --- | --- | --- |
| **Clinical characteristics at breast cancer diagnosis** |  |  |  |
| Age at diagnosis, years | 425 | 57.9 [49.6–66.6] | 53.2 [47.0–62.5] |
| Year of diagnosis | 425 | 2001 [1996–2007] | 2000 [1994–2006] |
| **Reproductive host factors^a^** |  |  |  |
| Parous at diagnosis | 411 | 230 (85.5%) | 130 (91.5%) |
| Number of births^b^ | 332 | 2 [2–3] | 2 [2–3] |
| Breastfeeding duration, months^b^ | 293 | 13 [7–23] | 14 [7–21] |
| Age at first birth, years^b^ | 297 | 23 [21–27] | 23 [21–27] |
| Years since first birth at diagnosis^b^ | 297 | 36 [27–46] | 30 [20–40] |
| **Pre-diagnostic host factors^c^** |  |  |  |
| **Clinical** |  |  |  |
| MAP (mmHg) | 379 | 94.5 [84.8–104.3] | 90.0 [84.7–100.7] |
| BMI (kg/m^2^) | 377 | 24.2 [22.1–27.2] | 23.3 [21.3–25.9] |
| **Timing of exposure assessment** |  |  |  |
| Years between the last survey cycle and diagnosis | 379 | 5.1 [2.5–10.0] | 5.4 [3.1–10.6] |
| **Tumor characteristics** |  |  |  |
| **Histologic type** | 421 |  |  |
| Invasive carcinoma NST |  | 217 (78.9%) | 123 (84.2%) |
| Invasive lobular carcinoma |  | 44 (16.0%) | 14 (9.6%) |
| Other |  | 14 (5.1%) | 9 (6.2%) |
| **Histological grade** | 353 |  |  |
| 1 |  | 82 (35.0%) | 32 (26.9%) |
| 2 |  | 122 (52.1%) | 51 (42.9%) |
| 3 |  | 30 (12.8%) | 36 (30.3%) |
| **T-status** | 400 |  |  |
| pT1 |  | 154 (58.8%) | 71 (51.4%) |
| pT2 |  | 87 (33.2%) | 58 (42.0%) |
| pT3-4 |  | 21 (8.0%) | 9 (6.5%) |
| **N-status** | 409 |  |  |
| pN0 |  | 192 (71.4%) | 85 (60.7%) |
| pN1 |  | 57 (21.2%) | 44 (31.4%) |
| pN2-3 |  | 20 (7.4%) | 11 (7.9%) |
| **Stage** | 422 |  |  |
| I |  | 133 (48.4%) | 60 (40.8%) |
| II |  | 116 (42.2%) | 71 (48.3%) |
| III |  | 26 (9.5%) | 16 (10.9%) |
| **Ki-67** | 384 |  |  |
| <20% |  | 192 (76.8%) | 72 (53.7%) |
| ≥20% |  | 58 (23.2%) | 62 (46.3%) |
| **HER2 status** | 411 |  |  |
| Negative |  | 238 (81.1%) | 105 (74.5%) |
| Positive |  | 32 (11.9%) | 36 (25.5%) |
| **HR status** | 425 |  |  |
| HR+ |  | 242 (87.1%) | 99 (67.3%) |
| HR– |  | 36 (12.9%) | 48 (32.7%) |

Continuous variables are presented as median [IQR], and categorical variables as n (%). n may vary across characteristics due to missing data.^a^Reproductive variables were derived using all available information across Tromsø Study surveys (Tromsø2–7): Information on age at births and breastfeeding was available from Tromsø4 onwards. Parity was supplemented with medical records, increasing completeness from 370 to 411 women. ^b^Parous women only.^c^Lifestyle and clinical variables were obtained from the most recent Tromsø Study survey cycle (Tromsø2–6) before breast cancer diagnosis.Abbreviations: BMI, body mass index; HR+, hormone receptor positive; HR–, hormone receptor negative; MAP, mean arterial pressure.

**References**

1.	Salgado R, Denkert C, Demaria S, et al. The evaluation of tumor-infiltrating lymphocytes (TILs) in breast cancer: recommendations by an International TILs Working Group 2014. *Annals of oncology* 2015; **26**(2): 259-71.
